# Chemical Composition and Insecticidal, Antiplasmodial, and Anti-Leishmanial Activity of *Capparis spinosa* Essential Oil and Its Main Constituents

**DOI:** 10.1155/2022/6371274

**Published:** 2022-02-01

**Authors:** Abeer Mousa Alkhaibari, Abdullah D. Alanazi

**Affiliations:** ^1^Department of Biology, Faculty of Science, University of Tabuk, Tabuk 71491, Saudi Arabia; ^2^Department of Biological Science, Faculty of Science and Humanities, Shaqra University, P.O. Box 1040, Ad-Dawadimi 11911, Saudi Arabia

## Abstract

**Background:**

This investigation was designed to evaluate the insecticidal, antiplasmodial, anti-leishmanial, and cytotoxic effects of *Capparis spinosa* essential oil (CSEO) and its main components, methyl isothiocyanate, hexadecanoic acid, and limonene.

**Methods:**

Insecticidal activity of CSEO and its main components, methyl isothiocyanate, hexadecanoic acid, and limonene, was determined against *Aedes aegypti* 4th-instar larvae at 25 ± 2°C. Antiplasmodial and anti-leishmanial effects of CSEO and its main components were carried out against chloroquine-resistant *Plasmodium falciparum* K1 strain and *Leishmania major* amastigotes based on the Malstat method and the macrophage model, respectively. We also performed the cytotoxic activity of CZEO and its main components against J774A1 macrophage cells using the colorimetric MTT (3-(4,5-dimethylthiazol-2-yl)-2,5-diphenyltetrazolium bromide) assay. In addition, the plasma membrane permeability and caspase-3-like activity CSEO and its main components were evaluated against *L. major*.

**Results:**

CSEO and its main components showed considerable (*p* < 0.001) larvicidal activity against *Ae. aegypti* larva. The 50% lethal concentration values for CSEO, methyl isothiocyanate, hexadecanoic acid, and limonene were 21.6, 30.9, 41.6, and 35.3 *μ*g/mL, respectively. By antiplasmodial effects, the 50% inhibitory concentration (IC_50_) values for CSEO, methyl isothiocyanate, hexadecanoic acid, and limonene were 7.4, 14.5, 19.6, and 21.3 *μ*g/mL, respectively, while these values for their anti-leishmanial effects were 9.1, 20.7, 23.3, and 18.6 *μ*g/mL, respectively. The 50% cytotoxic concentration values for CSEO, methyl isothiocyanate, hexadecanoic acid, and limonene were 93.7, 216.2, 199.4, and 221.3 *μ*g/mL, respectively. Different concentrations of CSEO and its main components significantly (*p* < 0.05) increased the plasma membrane permeability and caspase-3-like activity against *L. major* promastigote level as dose-dependent response.

**Conclusion:**

Based on the obtained results, *C. spinosa* essential oil and its main components, methyl isothiocyanate, hexadecanoic acid, and limonene, displayed insecticidal, antiplasmodial, and anti-leishmanial activity against healthy 4th-instar larvae of *A. aegypti*, chloroquine-resistant P. falciparum K1 strain, and L. major amastigotes, respectively. However, further surveys are required to display the mechanisms of action mode of tested drugs and their efficacy in animal model and clinical settings.

## 1. Introduction

Mosquitoes are considered as the main vectors of a wide range of important human infectious diseases including malaria, dengue, filariasis, encephalitis, and yellow fever, which cause high mortality around the world [1]. Among the mosquitoes important in medicine, *Aedes aegypti* L., as an anthropophilic and domicile mosquito, is broadly observed in the most tropical and subtropical regions worldwide [2]. This mosquito is well known as a vector of a number of important vector-borne infections such as malaria, dengue, chikungunya, Zika, and yellow fever [3].

Today, according to the recommendations of the World Health Organization (WHO), the main strategies for controlling vector are used as growth regulator insecticides such as diflubenzuron for immature forms of mosquitoes and the employment of some insecticides such as alpha-cypermethrin, malathion, and deltamethrin for controlling adult mosquitoes [4, 5]. In recent year, the excessive and constant use of these synthetic insecticides has led to a decrease in their efficacy and also some ecological worries such as emerging of drug resistance in mosquitoes, ecological imbalance, and outcome to animals [6, 7]. Hence, there is an rising concern to find new greatly selective and eco-friendly substitutes of insecticides around the world.

Malaria is a global public health problem, which estimated approximately 430,000 deaths annually, mostly in African children [8]. Although the most common *Plasmodium* species are *Plasmodium vivax* and *P. falciparum*, however, the most important species that causes high mortality in infected people is *P. falciparum* [9]. Meanwhile, the incidence of drug resistance in malaria, especially in *P. falciparum* malaria, is increasing to some synthetic antimalarial agents, such as chloroquine and mefloquine [10]. Thus, it is an urgent need for novel antimalarial agents, especially from natural resources with the least toxicity and the highest efficiency.

Leishmaniasis is one of the most significant protozoan infections triggered by the parasitic species *Leishmania,* which infects about 12 million people each year in 98 countries around the world [11]. *Leishmania* species in humans can cause cutaneous leishmaniasis, cutaneous-mucosal leishmaniasis, and visceral leishmaniasis [12]. A number of synthetic agents are applied to treat leishmaniasis; however, recent reviews have proven that most of the synthetic and chemical anti-leishmanial compounds have some limitations (e.g., drug resistance and long-term treatment), toxicity, and side effects [13].

Natural products and their derivatives as an inexpensive, accessible, and useful alternative medicine are broadly applied for the treatment of a wide range of diseases such as infectious ones [14]. Today, insecticides originating from plant extracts and essential oils have increased the overall interest, to replace or substitute synthetic insecticides [15]. Nowadays, there has been a growing development of research on the efficiency of medicinal herbs and their derivatives on various diseases, including parasitic diseases [14]. Although, in recent years, several laboratory and experimental studies have reported the significant antiparasitic effects of various medicinal herbs against *Leishmania* spp. and *Plasmodium* spp. 12,13, however, their efficacy and their toxicity are still debatable and vague [16].

*Capparis spinosa L.* (Capparaceae family) is an aromatic herb, which broadly grows in the Mediterranean area [17]. This plant is a multipurpose crop with unique properties in agro-biodiversity and agroecosystems such as resistance to drought and harsh environmental conditions. However, many biological aspects of this plant such as food and medicinal usages, phytochemistry, ethnopharmacology, and cultivation have not yet been studied [18].

In traditional medicine, *C. spinosa* was utilized as diuretic, appetizer, and anti-diarrheic and was used for the treatment of rheumatism, ulcers, ganglions, headache, and toothache [18, 19]. In addition to traditional uses, the *C. spinosa* has various ethnopharmacology and biological and chemical activity properties such as antioxidant, anticarcinogenic, anti-inflammatory, antidiabetic, and antimicrobial effects [18, 20]. The current investigation was designed to evaluate the insecticidal, antiplasmodial, anti-leishmanial, and cytotoxic effects of *C. spinosa* essential oil and its main components, methyl isothiocyanate, hexadecanoic acid, and limonene.

## 2. Materials and Methods

### 2.1. Compounds

In this study, methyl isothiocyanate, hexadecanoic acid, and limonene were obtained from Sigma-Aldrich (St. Louis, MO).

### 2.2. Plant Collection

*C. spinosa* aerial parts were provided in June 2021 from a market selling fresh herbs in Tabuk Market, Saudi Arabia, and they were identified by a botanist, and a voucher specimen (UT-2021-254) was deposited on the Herbarium of Department of Biology, Faculty of Science, University of Tabuk.

### 2.3. Preparing Essential Oil

To isolate the essential oil, 200 g of dried and powdered materials was put into the hydro-distillation technique for 180 min by means of a glass Clevenger-type device. The attained essential oil was then dehydrated by over anhydrous sodium sulfate and kept in darkness at 4°C in glass tubes until testing [21].

### 2.4. Gas Chromatography-Mass Spectrometry (GC-MS)

To recognize the compounds in CSEO, a Hewlett Packard 6890 (Palo Alto, CA, USA) device was used to perform the GC analysis equipped with a HP-5ms column (30 *m* × 0.25 mm, film thickness 0.25 mm). To do this, 0.1 *μ*L of essential oil was injected into the gas chromatography apparatus. The initial temperature was set at 50°C for 5 min and then increased to 300°C at a rate of 5°C/min. Helium gas was used at a rate of 1.1 mL/min, and ionization energy of electronvolt (EV) was used whereas the split ratio was 1:30, and injector and detector temperature was 280°C with split of 1/100. The components were identified according to the comparison of their mass spectra with those of NIST mass spectral library [22] and those explained by Adams and by comparison of their retention indices either with those of authentic compounds or with literature values [23].

### 2.5. Insecticidal Activity

Insecticidal activity of CSEO was performed according to the method explained by Huong et al. [24]. Briefly, *Ae. aegypti* eggs were prepared from the Department of Biology, Faculty of Science, University of Tabuk, Saudi Arabia, for further experiments. To do this, the various concentrations of CSEO and its main components, methyl isothiocyanate, hexadecanoic acid, and limonene (6.25, 12.5, 25, 50, and 100 *µ*g/mL), were dissolved in DMSO (1% stock solution) and were put in a 500-mL beaker and added to 150 ml water with 20 healthy 4th-instar larvae at 25 ± 2°C. Larval mortality was determined after 24 h of incubation; 50% lethal concentration (LC_50_) was calculated via the probit test in SPSS software for each drug. All tests were carried out in triplicate, and during experiments, no nutritional complement was added; however, DMSO was considered as the control group.

### 2.6. Antiplasmodial Activity

The antiplasmodial effects of various concentrations (3.125, 6.25, 12.5, 25, 50, and 100 *µ*g/mL) of CSEO and its main components, methyl isothiocyanate, hexadecanoic acid, and limonene, against chloroquine-resistant *P. falciparum* K1 strain were performed based on the Malstat method [25]. In brief, parasites were exposed to the human erythrocytes (red blood cells, RBC) in RPMI 1640 medium improved with 10% human serum and were incubated at 37°C with low oxygen atmosphere (3% O_2_, 4% CO_2_, and 93% N_2_). The infected human RBC (0.2 mL, 1% parasitaemia, and 2% hematocrit) was added to each well with various concentrations of CSEO and incubated for 3 days. Then, the tested plates were frozen at −20°C. In the next step, in a new plate, 0.1 mL of Malstat reagent was mixed with 0.02 mL of suspension of hemolyzed parasite and incubated for 15 min at 21°C. After this time, 0.02 Ml of NBT/PES solution was added to the plates and was incubated again for 120 min in the dark. Finally, the absorbance of each well was determined using the light absorption at 655 nm with the ELISA reader. The 50% inhibitory concentrations (IC_50_) were also calculated via the probit test in SPSS software.

### 2.7. Anti-Leishmanial Effects

To evaluate the anti-leishmanial effects of CSEO and its main components, methyl isothiocyanate, hexadecanoic acid, and limonene (3.125, 6.25, 12.5, 25, 50, and 100 *µ*g/mL), against the intracellular amastigote of *L. major* (MRHO/IR/75/ER), J774A1 macrophage cells (5 × 105 cells/ml) were poured in sterile 6-well plates (with 1 cm^2^ coverslips implanted on their floor) and incubated at 37°C for 24 hours with 5% CO_2_ to adhere to macrophages. After 24 hours, the plates were removed from the incubator and washed with sterile warm saline phosphate buffer. Then, 1 ml of RPMI 1640 enriched medium containing 5 × 10^6^ L. *major* promastigotes in the stationary phase was added into plates and kept warm at 37°C for 4 hours, and then, the wells were washed with RPMI 1640 medium to remove free promastigotes. In the next step, one ml of RPMI 1640 medium containing different concentrations of essential oil and MA was added to the wells for 48 hours, the slides were then fixed with methanol, and staining was then done with Giemsa dye diluted with water in a ratio of 1 : 10. The results were estimated by calculating the number of amastigotes inside 100 macrophages and the number of infected macrophages in each well. The IC_50_ values were also calculated via the probit test in SPSS software. All examinations in this study were carried out in triplicate [26].

### 2.8. Plasma Membrane Permeability

In this study, the effects of CSEO and its main components, methyl isothiocyanate, hexadecanoic acid, and limonene, on the permeability of plasma membrane of parasites were evaluated. To do this, *L. major* promastigotes of 1 × 10^6^ cells/ml were treated with different concentrations of CSEO, methyl isothiocyanate, hexadecanoic acid, and limonene (3.125, 6.25, and 12.5 *µ*g/mL) and then SYTOX Green stain was utilized based on the kit instructions. Parasites with no drug and those treated with 2.5% of Triton X-100 (Sigma-Aldrich) were determined as the negative control and positive control, respectively. The plasma membrane permeability was calculated by means of a microplate reader (BMG Labtech, Germany) for 4 h [26].

### 2.9. Evaluating the Caspase-3-Like Activity of Extract-Treated Promastigotes

The effects of CSEO, methyl isothiocyanate, hexadecanoic acid, and limonene on the induction of apoptosis were evaluated by the colorimetric protease (Sigma, Germany) method according to the manufacturer recommendations. In this way, the caspase-3-like activity level was measured based on the rate of color spectrophotometric produced through the release of a molecule (pNA attached to the substrate) under the enzyme caspase-3 activity. In brief, the promastigotes (1 × 10^6^) were incubated with CSEO, methyl isothiocyanate, hexadecanoic acid, and limonene at the concentrations of 6.25, 12.5, and 25 *µ*g/mL for 24 h and were centrifuged at 700 rpm for 5 minutes at 4°C. Next, the cell residue was lysed, and the cell lysate was centrifuged again at 20,000 rpm for 10 minutes. Lastly, the supernatant of reaction (5 *μ*l) was added to the 85 *μ*l of buffer and 10 *μ*l of caspase-3 (pNA-DEVD-Ac) solution and the mixture was incubated for 120 min at 37°C. The caspase-3-like activity was determined through the light absorption at 405 nm with the ELISA reader [27].

### 2.10. Cytotoxic Effects

We determined the cytotoxic activity of CSEO and its main components, methyl isothiocyanate, hexadecanoic acid, and limonene, against J774A1 macrophage cells, using the colorimetric MTT (3-(4,5-dimethylthiazol-2-yl)-2,5-diphenyltetrazolium bromide) assay based on the method described elsewhere. To perform it, the J774A1 macrophage cells (5 × 10^5^) were treated with various concentrations of CSEO (0 to 200 *μ*l/mL) and its main components, methyl isothiocyanate, hexadecanoic acid, and limonene (25, 50, 100, 200, and 400 *µ*g/mL), at the concentrations for 48 h in microplates at 37°C with 5% CO_2_. The 50% cytotoxic concentration (CC_50_) values were calculated by means of the probit test in SPSS software [26].

### 2.11. Statistical Analysis

To analyze the results, we used the SPSS statistical package, version 22.0 (SPSS, Inc.). To compare the results among tested groups, we applied the unpaired samples *t*-test and one-way analysis of variance (ANOVA), and Dunnett's test. *p* < 0.05 was considered statistically significant.

## 3. Results and Discussion

Medicinal herbs have been applied for centuries as appreciated resources of bioactive and beneficial materials with medical, industrial, and agricultural goals [28]. Herbal medicines, and their products such as essential oils and extracts, have been assessed for several strategies in the pest control and ovicidal activity and by evaluating the repellent [29]. In recent years, the present insecticidal agents commonly originated from a single active component, and herbal insecticides comprising combinations of chemical ingredients may affect both behavioral and physiological routes [30]. It seems that looking for bioinsecticides, which are effective, and being appropriate and adaptive to ecological situations, is necessary for gaining adequate insect control [31]. Essential oils are recognized to be complex combinations of secondary metabolites that may be acquired at low costs by means of updated technology, frequently demonstrating higher activities than the single isolated ingredients [32]. The current investigation was designed to evaluate the insecticidal, antiplasmodial, anti-leishmanial, and cytotoxic effects of *C. spinosa* essential oil. We found that the essential oil yielded 1.13% w/v; as shown in Table 1 of the obtained results in GC/MS, thirty-four compounds were identified, demonstrating 96.4% of the entire essential oil. The major components were methyl isothiocyanate (31.6%), hexadecanoic acid (18.5%), and limonene (11.6%), respectively, whereas the most chemical classes were aldehyde (48.4%), sesquiterpenes (19.4%), and monoterpene (12.9%), respectively.

In line with our results, Kulisic Bilusic (2010) has demonstrated that methyl isothiocyanate (92.06%) is the main constituent of essential oil of aerial parts of *C. spinosa* [33]. Ramdani et al. have reported that *C. spinosa* produced low yield (0.03%), whereas the main constituents of *C. spinosa* leaf essential oils obtained from six places in Algeria were hexadecanoic acid (38.19%), nonanal-n (12.61%), and cymene-2,5-dimethoxy-para (8.94%), respectively [34]. On the other hand, the results of the study conducted by Al-Mnaser demonstrate that the main constituents of the *C. spinosa* leaf essential oils are thymol (17%), octanoic acid (16%), methyl isothiocyanate (12%), and 2-hexenal (8.23%), respectively [35]. Esmaeilzadeh Bahabadi and Najafi have also reported that thymol (24%) and isothiocyanates (29%) are the most components of *C. spinosa* leaf essential oils [36]. Based on the previous reports, the chemical composition of essential oils is relatively different depending on several reasons including the place where the plant grew, the part of the herbs that is used, time of harvesting the herbs, and the technique of isolating the essential oil from the herbs [37, 38].

**Figure 1:**
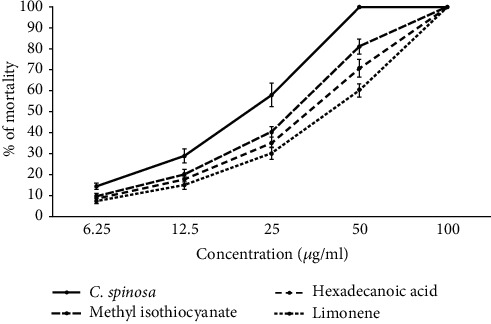
Insecticidal activity of the essential oil of *C. spinosa* and its main components, methyl isothiocyanate, hexadecanoic acid, and limonene, against *Ae. aegypti* larva. The results showed that among the main components of CSEO, methyl isothiocyanate and hexadecanoic acid displayed the highest and lowest larvicidal effects against *Ae. aegypti* larva. Data are presented as the mean ± SD (*n* = 3).

As shown in Figure 1, CSEO and its main components showed considerable (*p* < 0.001) larvicidal activity against *Ae. aegypti* larva. Based on the obtained LC_50_ results, the larvicidal effects of the components were found as CSEO > methyl isothiocyanate > limonene > hexadecanoic acid against *Ae. aegypti* larva, with the LC_50_ values for CSEO, methyl isothiocyanate, hexadecanoic acid, and limonene as 21.6, 30.9, 35.3, and 41.6, *μ*g/mL, respectively (Table 2).

Due to the lack of a unique and specific standard criterion in the guidelines of the WHO for evaluating the larvicidal activity of medicinal herbs, a number of researchers have established specific criteria to illustrate the efficacy of insecticides originated from herbal medicines [39, 40]. Based on the study conducted by Komalamisra et al. [41], medicinal herbs with LC_50_ value of less than 50  *µ*g/mL are promising and active; the products with LC_50_ values between 500 and100 *µ*g/mL were moderately active, whereas medicinal herbs with LC_50_ values between 100 and 750 *µ*g/mL were effective and those with LC_50_ values higher than 750 *µ*g/mL were inactive. The other study conducted by Kiran et al. demonstrated that natural products with LC_50_ less than 100 µg/mL are considered as a potent larvicidal effect [42]. It should be noted, however, that these criteria are dependent on exposure duration and larval source, which may alter the LC_50_ values of natural compounds examined [43]. Therefore, our finding revealed the promising and potent insecticidal effects of CSEO, based on the criterion reported by Komalamisra et al. [41] and Ravi Kiran et al. [42].

**Figure 2:**
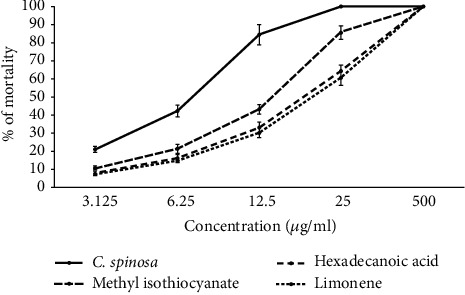
Antiplasmodial effects of various concentrations of CSEO and its main components, methyl isothiocyanate, hexadecanoic acid, and limonene, against chloroquine-resistant *P. falciparum* K1 strain based on the Malstat method. Among the main components of CSEO, the highest to the lowest antiplasmodial effect was observed in methyl isothiocyanate > hexadecanoic acid > and limonene. Data are presented as the mean ± SD (*n* = 3).

**Figure 3:**
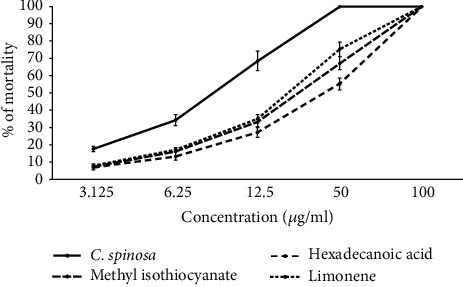
Anti-leishmanial effects of various concentrations of CSEO and its main components, methyl isothiocyanate, hexadecanoic acid, and limonene, against intracellular amastigote forms of *L. major* by the macrophage model, whereas among the main components of CSEO, the highest to the lowest anti-leishmanial effect was observed in limonene > methyl isothiocyanate > and hexadecanoic acid. Data are presented as the mean ± SD (*n* = 3).

The essential oil of *C. spinosa* and its main components, methyl isothiocyanate, hexadecanoic acid, and limonene, exhibited relevant (*p* < 0.001) effects against *P. falciparum* (Figure 2). The IC_50_ value for CSEO was 7.4 *μ*g/mL. Among the main components of CSEO, the highest to the lowest antiplasmodial effect was observed in methyl isothiocyanate > hexadecanoic acid > and limonene, with IC_50_ values of 14.5, 19.6, and 21.3 *μ*g/mL, respectively (Table 2). As shown in Figure 3, the essential oil of *C. spinosa* and its main components, methyl isothiocyanate, hexadecanoic acid, and limonene, displayed activity property (*p* < 0.001) against intracellular amastigotes of *L. major*. The IC_50_ value for CSEO was 9.1 *μ*g/mL; based on the obtained LC_50_ values, the anti-leishmanial effects of the main components were found as limonene > methyl isothiocyanate > and hexadecanoic acid, with IC_50_ values of 18.6, 20.7, and 23.3 *μ*g/mL, respectively (Table 2).

**Figure 4:**
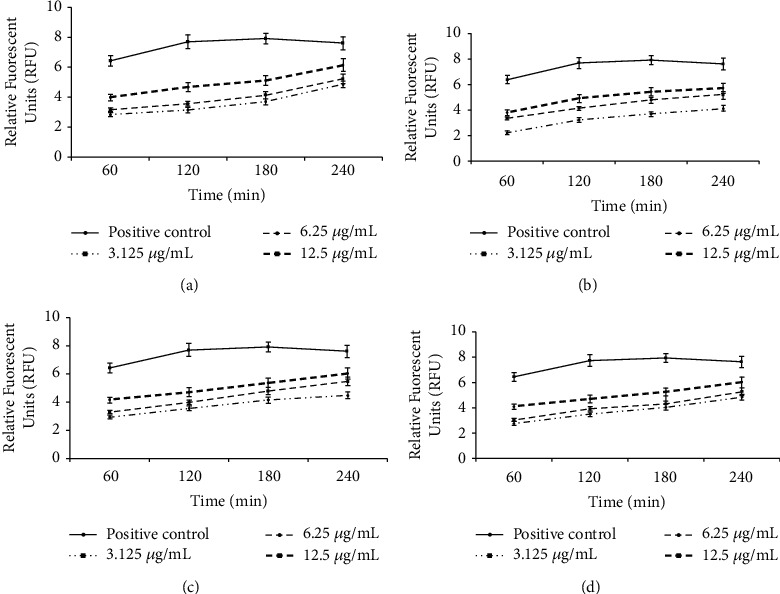
Plasma membrane permeability of the *L. major* promastigotes treated with CSEO (a), hexadecanoic acid (b), methyl isothiocyanate (c), and limonene (d). The results that exhibited relative fluorescent units revealed that the promastigotes treated with CSEO, methyl isothiocyanate, hexadecanoic acid, and limonene as dose-dependent response changed the permeability of plasma membrane by SYTOX Green. Data are presented as the mean ± SD (*n* = 3).

Previous investigations have demonstrated that the rupture and/or cross-plasma membrane is recognized as one of the main action modes to inhibit the growth of intracellular pathogens [25]; therefore, we evaluated the plasma membrane permeability of the *L. major* promastigotes treated with CSEO, methyl isothiocyanate, hexadecanoic acid, and limonene. The findings of relative fluorescent units demonstrated that the promastigotes treated with CSEO, methyl isothiocyanate, hexadecanoic acid, and limonene changed the permeability of plasma membrane by SYTOX Green as dose-dependent response (Figure 4).

**Figure 5:**
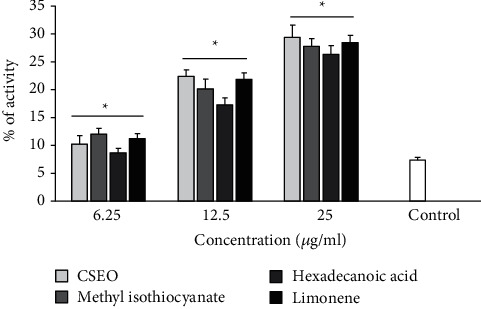
Caspase-3-like activity of *L. major* promastigotes treated with CSEO, methyl isothiocyanate, hexadecanoic acid, and limonene using the colorimetric protease methods. The results exhibited that CSEO, methyl isothiocyanate, hexadecanoic acid, and limonene significantly induced caspase-3 activation as dose-dependent response. ^*∗*^*p* < 0.05 shows that the difference was statistically significant in comparison with control. Data are presented as the mean ± SD (*n* = 3).

Apoptosis is considered as a form of programmed cell death process that happens in multicellular organisms. The process is a complex cascade of protease pathways designed to cellular death in a selective mode [44]. Among the main mediators of apoptosis, caspases and especially caspase-3 are considered as one of the crucial caspases that principally activated apoptosis-related proteases and consecutively prompt cell death [45]. The findings of the present investigation exhibited that we found that CSEO, methyl isothiocyanate, hexadecanoic acid, and limonene significantly induced caspase-3 activation as dose-dependent response ranging from 8.6 to 29.3% in comparison with the control (Figure 5).

We found that the main components of CSEO are organosulfur (methyl isothiocyanate), fatty acid (hexadecanoic acid), and monoterpenoid (limonene) compounds. Considering the antimicrobial mechanisms of organosulfur compounds, previous investigations reported that these compounds displayed their antimicrobial mechanisms through disruption of DNA, RNA, and protein synthesis, reacting with sulfhydryl groups of the enzymes and proteins of microbes, damaging the cell wall and membrane, and subsequently integrity of cell membrane integrity [46–49]. Fatty acids also showed their antimicrobial mechanisms through the cell membrane damage, by disruption of the electron transport chain and oxidative phosphorylation of microbes [50]. With respect to the antimicrobial action mode of monoterpenoid, previous reports exhibited that these compounds affect cell membrane permeability of microbes and interact with intracellular targets [51–53].

Babanezhad Harikandei et al. (2020) have demonstrated that the synthesized isothiocyanate derivatives significantly reduced the growth rate of *Trypanosoma brucei rhodesiense* strain STIB900 (with IC_50_ values ranging from 1 to 46.6 *µ*g/mL), *T. cruzi* strain Tulahuen C4 (with IC_50_ values ranging from 1.9 to 10.6 *µ*g/mL), *L. donovani* axenic amastigote strain MHOM-ET-67/L82 (with IC_50_ values ranging from 0.4 to 7.1 *µ*g/mL), and *P. falciparum* strain NF54 (with IC_50_ values ranging from 1.1 to 10.3 *µg*/mL) [54]. Hamdi et al. (2018) have reported that limonene significantly reduced and killed the *L. mexicana* promastigotes with IC_50_ value of 16.59 *µ*g/mL [55]. Wang et al. have demonstrated the insecticidal activity of limonene against *Tribolium castaneum* (Herbst) and *Lasioderma serricorne* (Fabricius) with LC_50_ values of 14.97 and 13.66 *μ*g/mL, respectively [56]. Hence, it may be proposed that in addition to the high activity of individual compounds the activity of essential oil is influenced by the synergism of the compounds in lower concentration.

**Figure 6:**
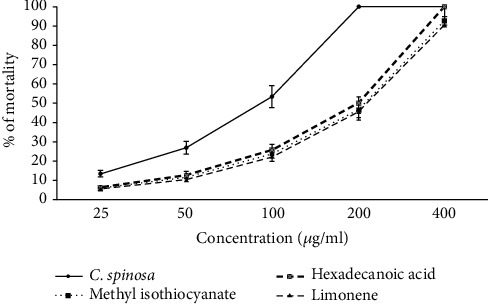
Cytotoxicity effects of various concentrations of CSEO and its main components, methyl isothiocyanate, hexadecanoic acid, and limonene against J774A4 macrophage cells by the MTT assay. Data are presented as the mean ± SD (*n* = 3).

The results of MTT assay in this study exhibited that the essential oil of *C. spinosa* and its main components, methyl isothiocyanate, hexadecanoic acid, and limonene, showed no significant cytotoxicity against macrophage cells (Figure 6). The CC_50_ values for CSEO, methyl isothiocyanate, hexadecanoic acid, and limonene, were 93.7, 216.2, 199.4, and 221.3 *μ*g/mL, respectively (Table 3). Previously, Kulisic Bilusic et al. (2012) have revealed that the essential oil of *C. spinosa* collected from Split City, Croatia, reduced the displayed cytotoxic effects against the HT-29 cells with the CC_50_ values of 261.3 and 373.6 *µ*g/mL, respectively [57].

## 4. Conclusion

Based on the obtained results, *C. spinosa* essential oil and its main components, methyl isothiocyanate, hexadecanoic acid, and limonene, displayed insecticidal, antiplasmodial, and anti-leishmanial activity against healthy 4th-instar larvae of *A. aegypti*, chloroquine-resistant *P. falciparum* K1 strain, and *L. major* amastigotes, respectively. In addition, the various concentrations of CSEO, methyl isothiocyanate, hexadecanoic acid, and limonene significantly (*p* < 0.05) increased the plasma membrane permeability and caspase-3-like activity level as dose-dependent response with no consideration of the cytotoxicity against J774A1 macrophage cells. However, further surveys are required to display the mechanisms of action mode of tested drugs and their efficacy in animal model and clinical settings.

## Figures and Tables

**Table 1:** GC/MS analysis of *C. spinosa* essential oil

No.	Compound	RI_C_	RI_L_	Area (%)
1.	Tert-butanol	676	669	0.6
2.	Methyl isothiocyanate	703	704	31.6
3.	Cyclohexane	712	717	0.3
4.	Hexanal	802	804	0.3
5.	Heptanal	892	894	2.1
6.	Butyl isothiocyanate	947	959	1.1
7.	*β*-Pinene	974	980	1.2
8.	Octanal-n	1003	1001	1.1
9.	p-Cymene	1026	1027	0.7
10.	Limonene	1030	1036	11.6
11.	*β*-Phellandrene	1034	1043	0.8
12.	Benzeneacetaldehyde	1043	1044	0.5
13.	Octen-1-ol-2e	1064	1066	1.3
14.	Nonanal-n	1102	1102	7.6
15.	Benzeneacetonitrile	1140	1145	0.8
16.	Methyl salicylate	1198	1195	0.9
17.	Decenal-z-4	1263	1265	1.6
18.	Decanol-n	1272	1279	1.8
19.	Tetradecane	1400	1393	0.5
20.	*β*-Caryophyllene	1417	1421	0.7
21.	l-Octen-3-ol	1456	1458	0.6
22.	2-Tridecanone	1492	1497	0.7
23.	Pentadecane	1501	1509	0.7
24.	Tridecanal	1510	1512	0.6
25.	Germacrene B	1551	1556	0.4
26.	3-Hexenyl benzoate	1566	1558	0.6
27.	Caryophyllene oxide	1581	1580	0.5
28.	Tetradecanal	1613	1611	1.0
29.	Hexadecanoic acid	1962	1970	18.5
30.	Docosane	2211	2200	4.3
31.	Tetracosane-n	2416	2400	1.4
**Total**			**96.4**	

**Table 2:** Total content of compounds of *C. spinosa* essential oil by GC/MS analysis.

Chemical class	Percent (%)
Alcohol	9.6
Aldehyde	48.4
Monoterpene	12.9
Organosulfur	6.5
Sesquiterpenes	19.4
Fatty acid	3.2

**Table 3:** Insecticidal, antiplasmodial, and anti-leishmanial activity of the essential oil of *C. spinosa* and its main components, methyl isothiocyanate, hexadecanoic acid, and limonene.

Drug	Insecticidal activity LC_50_ (*μ*g/mL)	Anti-leishmanial effect IC_50_ (*μ*g/mL)	Antiplasmodial activity IC_50_ (*μ*g/mL)	Cytotoxicity effect CC_50_ (*μ*g/mL)
*C. spinosa* essential oil	21.6 ± 2.15	7.4 ± 0.89	9.1 ± 1.12	93.7 ± 4.54
Methyl isothiocyanate	30.9 ± 3.12	14.5 ± 2.51	20.7 ± 2.54	216.2 ± 8.65
Hexadecanoic acid	41.6 ± 3.62	19.6 ± 3.05	23.3 ± 3.14	199.4 ± 7.58
Limonene	35.3 ± 4.23	21.3 ± 2.15	18.6 ± 2.19	221.3 ± 9.87

## Data Availability

The data presented in this study are available in this article.

## References

[1] Brown M., Hebert A. A. (1997). Insect repellents: an overview. *Journal of the American Academy of Dermatology*.

[2] Souza-Neto J. A., Powell J. R., Bonizzoni M. (2019). *Aedes aegypti* vector competence studies: a review. *Infection, Genetics and Evolution*.

[3] Powell J. R., Tabachnick W. J. (2013). History of domestication and spread of *Aedes aegypti* - a Review. *Memórias do Instituto Oswaldo Cruz*.

[4] Sutthanont N., Attrapadung S., Nuchprayoon S. (2019). Larvicidal activity of synthesized silver nanoparticles from Curcuma zedoaria essential oil against *Culex quinquefasciatus*. *Insects*.

[5] Chareonviriyaphap T., Bangs M. J., Suwonkerd W., Kongmee M., Corbel V., Ngoen-Klan R. (2013). Review of insecticide resistance and behavioral avoidance of vectors of human diseases in Thailand. *Parasites & Vectors*.

[6] Pavela R. (2008). Larvicidal effects of various Euro-Asiatic plants against *Culex quinquefasciatus* Say larvae (Diptera: Culicidae). *Parasitology Research*.

[7] Abdul Rahuman A., Gopalakrishnan G., Venkatesan P., Geetha K. (2008). Isolation and identification of mosquito larvicidal compound from Abutilon indicum (Linn.) Sweet. *Parasitology Research*.

[8] Bannister-Tyrrell M., Verdonck K., Hausmann-Muela S., Gryseels C., Ribera J. M., Grietens K. P. (2017). Defining micro-epidemiology for malaria elimination: systematic review and meta-analysis. *Malaria Journal*.

[9] Sabina K. (2017). Prevalence and epidemiology of malaria in Nigeria: a review. *International Journal of Research in Pharmacy and Biosciences*.

[10] Newby G., Hwang J., Koita K. (2015). Review of mass drug administration for malaria and its operational challenges. *The American Journal of Tropical Medicine and Hygiene*.

[11] Kevric I., Cappel M. A., Keeling J. H. (2015). New world and old world Leishmania infections. *Dermatologic Clinics*.

[12] Albalawi A. E., Khalaf A. K., Alyousif M. S. (2021). Fe3O4@piroctone olamine magnetic nanoparticles: synthesize and therapeutic potential in cutaneous leishmaniasis. *Biomedicine & Pharmacotherapy*.

[13] Monzote L. (2009). Current treatment of leishmaniasis: a review. *The Open Antimicrobial Agents Journal*.

[14] Alnomasy S., Al-Awsi G. R., Raziani Y. (2021). Systematic review on medicinal plants used for the treatment of Giardia infection. *Saudi Journal of Biological Sciences*.

[15] Khare R. K., Das G., Kumar S. (2019). Herbal insecticides and acaricides: challenges and constraints. *International Journal of Chemical Studies*.

[16] Wink M. (2012). Medicinal plants: a source of anti-parasitic secondary metabolites. *Molecules*.

[17] Rahnavard R., Razavi N. (2017). A review on the medical effects of Capparis spinosa L. *Advanced Herbal Medicine*.

[18] Ebrahimi K., Shiravand S., Mahmoudvand H. (2017). Biosynthesis of copper nanoparticles using aqueous extract of Capparis spinosa fruit and investigation of its antibacterial activity. *Marmara Pharmaceutical Journal*.

[19] Rivera D., Inocencio C., Obón C., Alcaraz F. (2003). Review of food and medicinal uses of Capparis L. subgenus Capparis (Capparidaceae). *Economic Botany*.

[20] Nabavi S. F., Maggi F., Daglia M., Habtemariam S., Rastrelli L., Nabavi S. M. (2016). Pharmacological effects ofCapparis spinosaL. *Phytotherapy Research*.

[21] Shaapan R. M., Al-Abodi H. R., Alanazi A. D. (2021). Myrtus communis essential oil; anti-parasitic effects and induction of the innate immune system in mice with toxoplasma gondii infection. *Molecules*.

[22] Nist N. (2014). *EPA/NIH Mass Spectral Library*.

[23] Adams R. P. (2004). *Identification of Essential Oil Components by Gas Chromatography/mass Spectroscopy*.

[24] Huong L. T., Hung N. H., Dai D. N. (2019 Jan). Chemical compositions and mosquito larvicidal activities of essential oils from Piper species growing wild in Central Vietnam. *Molecules*.

[25] Makler M. T., Piper R. C., Williams J. A. (1993). Parasite lactate dehydrogenase as an assay for Plasmodium falciparum drug sensitivity. *The American Journal of Tropical Medicine and Hygiene*.

[26] Albalawi A. E., Abdel-Shafy S., Khudair Khalaf A. (2021). Therapeutic potential of green synthesized copper nanoparticles alone or combined with meglumine antimoniate (glucantime) in cutaneous leishmaniasis. *Nanomaterials*.

[27] Albalawi A. E. (2021). Antileishmanial activity of ziziphus spina-christi leaves extract and its possible cellular mechanisms. *Microorganisms*.

[28] Yuan H., Ma Q., Ye L., Piao G. (2016). The traditional medicine and modern medicine from natural products. *Molecules*.

[29] Asadollahi A., Khoobdel M., Zahraei-Ramazani A., Azarmi S., Mosawi S. H. (2019). Effectiveness of plant-based repellents against different Anopheles species: a systematic review. *Malaria Journal*.

[30] Ghosh A., Chowdhury N., Chandra G. (2012). Plant extracts as potential mosquito larvicides. *Indian Journal of Medical Research*.

[31] Dayan F. E., Cantrell C. L., Duke S. O. (2009). Natural products in crop protection. *Bioorganic & Medicinal Chemistry*.

[32] Pavela R. (2015). Essential oils for the development of eco-friendly mosquito larvicides: a review. *Industrial Crops and Products*.

[33] Kulisic‐Bilusic T., Blažević I., Dejanović B., Miloš M., Pifat G. (2010). Evaluation of the antioxidant activity of essential oils from caper (Capparis spinosa) and sea fennel (Crithmum maritimum) by different methods. *Journal of Food Biochemistry*.

[34] Ramdani M., Lograda T., Chalard P. (2020). Chemical composition and antibacterial activities of Capparis spinosa essential oils from Algeria. *Biodiversitas Journal of Biological Diversity*.

[35] El-Naser Z. (2016). Analysis of essential oil of Capparis spinosa L. leaves and interaction between Pieris brassicae L.(Lepidopteran) which attack caper and natural enemy Cotesia glomerata (L.). *International Journal of ChemTech Research*.

[36] Esmaeilzadeh Bahabadi S., Najafi S. (2016). Essential oil composition and antioxidant optimization of Capparis spinosa L. Fruit in sistan region. *Eco-Phytochemical Journal of Medical Plants*.

[37] Mahmoudvand H., Kheirandish F., Ghasemi Kia M., Tavakoli Kareshk A., Yarahmadi M. (2016). Chemical composition, protoscolicidal effects and acute toxicity of Pistacia atlantica Desf. fruit extract. *Natural Product Research*.

[38] Saedi Dezaki E., Mahmoudvand H., Sharififar F., Fallahi S., Monzote L., Ezatkhah F. (2016). Chemical composition along with anti-leishmanial and cytotoxic activity of Zataria multiflora. *Pharmaceutical Biology*.

[39] Chantraine J.-M., Laurent D., Ballivian C., Saavedra G., Ibañez R., Vilaseca L. A. (1998). Insecticidal activity of essential oils onAedes aegypti larvae. *Phytotherapy Research*.

[40] Magalhães L. A., Lima M. P., Marques M. O., Facanali R., Pinto A. C., Tadei W. P. (2010). Chemical composition and larvicidal activity against *Aedes aegypti* larvae of essential oils from four Guarea species. *Molecules*.

[41] Komalamisra N., Trongtokit Y., Rongsriyam Y., Apiwathnasorn C. (2005). Screening for larvicidal activity in some Thai plants against four mosquito vector species. *Southeast Asian Journal of Tropical Medicine & Public Health*.

[42] Ravi Kiran S., Bhavani K., Sita Devi P., Rajeswara Rao B. R., Janardhan Reddy K. (2006). Composition and larvicidal activity of leaves and stem essential oils of Chloroxylon swietenia DC against *Aedes aegypti* and *Anopheles stephensi*. *Bioresource Technology*.

[43] Dias C. N., Alves L. P., Rodrigues K. A. (2015). Chemical composition and larvicidal activity of essential oils extracted from Brazilian legal amazon plants against *Aedes aegypti* L. (Diptera: Culicidae). *Evidence-based Complementary and Alternative Medicine: eCAM*.

[44] Elmore S. (2007). Apoptosis: a review of programmed cell death. *Toxicologic Pathology*.

[45] Portt L., Norman G., Clapp C., Greenwood M., Greenwood M. T. (2011). Anti-apoptosis and cell survival: a review. *Biochimica et Biophysica Acta (BBA)-Molecular Cell Research*.

[46] Nakamoto M., Kunimura K., Suzuki J. I., Kodera Y. (2020). Antimicrobial properties of hydrophobic compounds in garlic: allicin, vinyldithiin, ajoene and diallyl polysulfides. *Experimental and Therapeutic Medicine*.

[47] Ankri S., Mirelman D. (1999). Antimicrobial properties of allicin from garlic. *Microbes and Infection*.

[48] Sagdic O., Tornuk F. (2012). Antimicrobial properties of organosulfur compounds. *Dietary Phytochemicals and Microbes*.

[49] Bhatwalkar S. B., Mondal R., Krishna S. B., Adam J. K., Govender P., Anupam R. (2021). Antibacterial properties of organosulfur compounds of garlic (allium sativum). *Frontiers in Microbiology*.

[50] Desbois A. P., Smith V. J. (2010). Antibacterial free fatty acids: activities, mechanisms of action and biotechnological potential. *Applied Microbiology and Biotechnology*.

[51] Sikkema J., de Bont J. A., Poolman B. (1995). Mechanisms of membrane toxicity of hydrocarbons. *Microbiological Reviews*.

[52] Ismail A., Lamia H., Mohsen H., Samia G., Bassem J. (2013). Chemical composition and antifungal activity of three Anacardiaceae species grown in Tunisia. *Science International*.

[53] Mahmoudvand H., Khalaf A. K., Beyranvand M (2021). In Vitro and ex vivo evaluation of Capparis spinosa extract to inactivate protoscoleces during hydatid cyst surgery. *Current Drug Discovery Technologies*.

[54] Babanezhad Harikandei K., Salehi P., Ebrahimi S. N., Bararjanian M., Kaiser M., Al-Harrasi A. (2020). Synthesis, in-vitro antiprotozoal activity and molecular docking study of isothiocyanate derivatives. *Bioorganic & Medicinal Chemistry*.

[55] Hamdi A., Bero J., Beaufay C. (2018). In vitro antileishmanial and cytotoxicity activities of essential oils from Haplophyllum tuberculatum A. Juss leaves, stems and aerial parts. *BMC Complementary and Alternative Medicine*.

[56] Wang Y., You C.-X., Wang C.-F. (2014). Chemical constituents and insecticidal activities of the essential oil from Amomum tsaoko against two stored-product insects. *Journal of Oleo Science*.

[57] Kulisic-Bilusic T., Schmöller I., Schnäbele K., Siracusa L., Ruberto G. (2012). The anticarcinogenic potential of essential oil and aqueous infusion from caper (Capparis spinosa L.). *Food Chemistry*.

